# Contribution from MHC-Mediated Risk in Schizophrenia Can Reflect a More Ethnic-Specific Genetic and Comorbid Background

**DOI:** 10.3390/cells11172695

**Published:** 2022-08-30

**Authors:** Lekshmy Srinivas, Neetha N. Vellichirammal, Indu V. Nair, Chandrasekharan M. Nair, Moinak Banerjee

**Affiliations:** 1 Human Molecular Genetics Laboratory, Rajiv Gandhi Centre for Biotechnology, Trivandrum 695014, India; 2 Mental Health Centre, Trivandrum 695005, India; 3 Nair’s Hospital, Kerala 682304, India

**Keywords:** schizophrenia, MHC, SNPs, immune system, environment, psoriasis, genetics

## Abstract

The immune system seems to play a significant role in the development of schizophrenia. This becomes more evident with the emerging role of MHC complex and cytokines in schizophrenia. In the recent past, several GWAS have implied that the 6p21 region was associated with schizophrenia. However, the majority of these studies were performed in European populations. Considering tremendous variations in this region and the probability of South Indian populations being quite different from the European gene-pool from an immunogenetic point, the present study was initiated to screen SNPs in the 2.28 MB region, spanning the extended MHC locus, in 492 cases and controls from a South Indian population. We found a very strong association of rs3815087 with schizophrenia at both allelic and genotypic levels with a 7.3-fold increased risk in the recessive model. Interestingly, the association of none of the earlier reported GWAS hits, such as rs3130375, rs3131296, rs9272219, or rs3130297 were found to be replicable in our study population. rs3815087 lies in the 5′UTR region of the psoriasis susceptibility 1 candidate 1 (*PSORS1C1*) gene, which further suggests that inflammatory processes might be an important common pathogenic pathway leading to both schizophrenia and psoriasis. The study hints at ethnic specific gene–environment interaction in determining the critical threshold for disease initiation and progression.

## 1. Introduction

The focus of schizophrenia research has been turning away from the pathophysiology of neurotransmission to possibly more heterogeneous etiological factors. Investigations based on personal history data indicate that auto-immune diseases are associated with 45% increased risk for developing schizophrenia. Among these, 9 auto-immune disorders had higher prevalence rates among patients and 12 auto-immune diseases were significantly more prevalent among the relatives of patients with schizophrenia [1]. In the early 1960s, several investigators described a variety of antibrain antibodies in the sera of patients with schizophrenia [2,3]. Several groups have reported the increased occurrence of autoantibodies in people with schizophrenia, when compared to controls, against the brain or specific areas of the brain (including the cerebrum) [4], septum [5] and amygdala, frontal cortex, cingulate gyrus, and septal area [6] or against brain constituents such as gangliosides [7]. Aberrant activation of humoral immunity, innate immunity, and autoimmunity have been reported to be associated with schizophrenia [8,9]. In light of all these observations, it is highly probable that the immune hypothesis of schizophrenia fits well with the supposed interaction between genetic and environmental factors.

The key regulators of immunity and inflammation that are involved in the neurobiological processes related to neurodevelopment, neuronal plasticity, learning, memory, and behavior are cytokines and MHC. While cytokines had been the point of intense investigation [10,11], three GWAS and the meta-analysis of GWAS data in 2009 shifted the focus to the MHC region by providing definitive evidence of the involvement of MHC in schizophrenia [12,13,14]. This was further validated in a Swedish cohort and Psychiatric Genomics Consortium data [15]. The role of MHC in schizophrenia was first reviewed by McGuffin in 1979 [16], who also postulated the autoimmune pathogenesis. Since then, several studies have implied the role of HLA in various ethnic populations [17,18,19]. The highly diverse nature of the MHC region across ethnicities prompted us to investigate whether this reported association of SNPs in the MHC region holds true in the South Indian population.

## 2. Materials and Methods

### 2.1. Subject Selection

A case-control association study was carried out using 248 schizophrenia patients (98 males and 146 females) with mean age group of 33 ± 11 years and 244 controls (83 males and 161 females) with mean age group of 33 ± 7 years that belonged to an ethnically matched Malayalam-speaking population of Kerala, South India. These samples were genetically [20] and epigenetically [21] stratified based on their ethnicity as assessed by their genetic architecture and similar environmental background. Patients were diagnosed as per the Diagnostic and Statistical Manual of Mental Disorders, 4th Edition (DSM-IV) criteria for schizophrenia by two psychiatrists who were part of the study. Patients with major affective disorders and schizo-affective disorder were excluded. Patients with narcotic drugs or alcohol abuse were also excluded. All the participants who consented were included in the study. The study was approved by the Institutional Ethics Committee for Biomedical Subjects (IHEC/01/2011/01), according to the Indian Council of Medical Research (ICMR) guidelines. Peripheral blood was collected and used for DNA isolation.

### 2.2. MHC Region SNP Selection

SNPs from a 2.28 M bp region (30429711–32710247) spanning the extended MHC in chromosome 6p21.3 were selected to test for association with schizophrenia in the Kerala population. This also includes the top three SNPs, namely, rs3130375, rs3131296, rs9272219 [12,13,14,15] and imputed the SNP, rs3130297, proximal to *NOTCH4* gene [22] which has also been associated with schizophrenia in European ancestry. A list of the selected SNPs, their chromosomal positions, nearby genes, and predicted functional effects are given in Table 1. Functional effect predictions of SNPs were done using Functional SNP Prediction tool, FuncPred (snpinfo.niehs.nih.gov/snpinfo/snpfunc.htm accessed on 25 July 2022) and FASTSNP (fastsnp.ibms.sinica. edu.tw accessed on 25 July 2022). Genomic DNA was isolated and SNP genotyping was carried out by real time based TaqMan allelic discrimination assay (Applied Biosystems, Foster City, CA, USA) and Competitive Allele-Specific PCR genotyping system (KASPar) assay (KBiosciences, Hoddesdon, EN, UK) according to manufacturers’ instructions in an Applied Biosystems 7500 Real-Time PCR. Gene expression for the allelic variants were evaluated using GTex data portal (gtexportal.org).

### 2.3. Statistical Analyses

Allelic and genotypic frequencies were calculated and compared using Unphased v3.0.13 software (Cambridge, UK). Odds ratios (OR), as the estimates of relative risk of disease, with 95% confidence intervals, were calculated to compare allele and genotype frequencies using Chi-square analysis. The *p*-values of the SNPs were corrected for false discovery rate (FDR) using the Benjamini–Hochberg method for multiple correction. Association was also analyzed using dominant, recessive, and additive genetic models for risk genotypes, and only the significant models are presented. Deviations from the Hardy–Weinberg equilibrium (HWE) were tested for all polymorphisms in controls by comparing observed and expected genotype frequencies (ihg2.helmholtz-muenchen.de/cgi-bin/hw/hwa1.pl accessed on 25 July 2022). The LD structure among SNPs was visualized by generating LD plots using the Haploview v4.1 program (www.broad.mit.edu/mpg/haploview/ accessed on 25 July 2022). The degree of LD between pairs of loci was estimated using r^2^ values (correlation coefficient). A triangular matrix of r^2^ value was used to demonstrate LD patterns within cases and controls. 

## 3. Results

### Association of SNPs in the MHC Region with Schizophrenia

**Figure 1:**
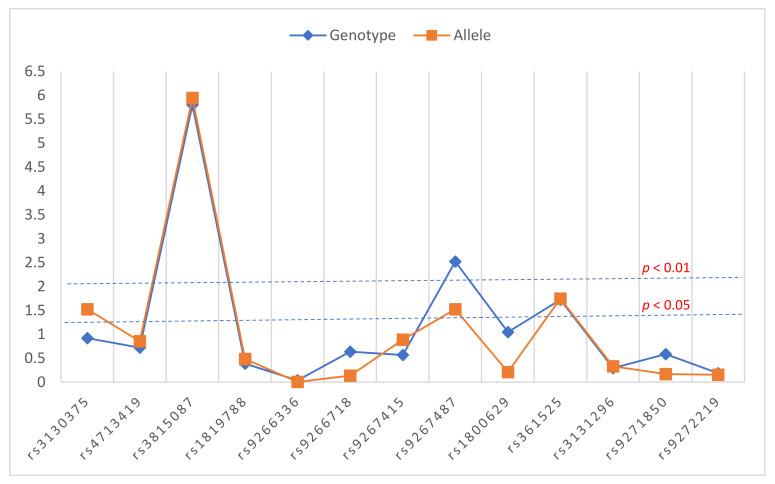
Association of SNPs in MHC region with schizophrenia. X-axis shows the polymorphisms screened and Y-axis shows the *p* values for each polymorphism on a logarithmic scale.

In the extended MHC region spanning 2.28 MB (30429711–32710247) of chromosome 6p21.3 region, 15 SNPs were screened for their association with schizophrenia in a Malayalam-speaking South Indian population from Kerala. Among these, two SNPs, rs9368649 (AA) and rs3130297 (CC), were found to be monomorphic in our population and were excluded from further analysis. There were no significant deviations from heterozygosity values expected under HWE for the studied SNPs in controls with exception to rs9271850. We found association of multiple SNPs in the MHC region associated with schizophrenia susceptibility (Figure 1, Table 2). The most significant association was observed for SNP rs3815087. The T allele (OR = 2, *p* = 1.14 × 10^−6^) and TT genotype (*p* = 1.58 × 10^−6^) were observed to be very strongly associated with schizophrenia. In the recessive model, the TT genotype conferred a 7.3-fold increased risk for schizophrenia. Significant association with schizophrenia was also observed for rs9267487 at the allelic (*p* = 0.03, OR = 1.66) and genotypic (*p* = 0.003) levels. SNP rs361525 allele and genotypes were also observed to be associated with schizophrenia (*p* = 0.018 and *p* = 0.019, respectively). The association of rs3815087 remained highly significant even after correcting for multiple comparisons, both at allelic (FDR corrected *p* = 1.48 × 10^−5^) and genotypic levels (FDR corrected *p* = 2.05 × 10^−5^) while for other SNPs, this association was lost after multiple corrections. From a combined meta-analysis of *p*-values from ISC, MGS, and SGENE cohorts and our present data, we observe that rs3815087 reaches a genome wide significance of *p* = 1.90 × 10^−8^.

## 4. Discussion

While trying to replicate the top GWAS hits from the MHC region reported in the European population, we found a contrasting association in Malayalam-speaking South Indian population from Kerala. We did not find any association of the top three SNPs reported by GWAS, rs3130375, rs3131296, or rs9272219, with schizophrenia in our Kerala population. Another SNP rs3130297, the best imputed SNP from the ISC study, was found to be monomorphic in our population. Interestingly, we found a strong association of rs3815087, rs9267487, and rs361525 with schizophrenia at the allelic and genotypic levels. The presence of risk TT genotype of rs3815087 conferred a 7.3-fold increased risk for schizophrenia (*p* = 1.17 × 10^−6^, recessive model TT vs. CT+CC). The T allele was also strongly associated with the disease (OR = 2, *p* = 1.14 × 10^−6^). The association of rs3815087 remained highly significant even after correcting for multiple comparisons. This SNP shows significant variation across ethnicities in the South Asian population displaying the highest minor allele frequency, which is different from the Europeans.

The SNP conferring the strongest risk, rs3815087, lies in the 5′UTR region of the psoriasis susceptibility 1 candidate 1 (*PSORS1C1*) gene. The *PSORS1C1* gene, located 127 kb telomeric to the HLA-C locus, is considered to be one of the potential candidate genes for psoriasis. The SNP rs3815087 is reported to have a role in splicing regulation. rs3815087 was not in linkage disequilibrium with nearby SNPs. This SNP rs3815087 was analyzed in three GWA studies conducted by three groups (ISC, MGS, and SGENE) published jointly in Nature in 2009 [12,13,14,15]. Although no *p*-values reached genome-wide significance in the three independent cohorts, namely ISC (*p* = 0.077, OR = 1.09, 95% CI = 0.99–1.21), MGS (*p* = 0.22, OR = 1.06, 95% CI = 0.96–1.17,) and SGENE (*p* = 1.30 × 10^−4^, OR = 1.20, 95% CI = 1.09–1.31), the combined *p*-values reached genome-wide significance (*p* = 6.7 × 10^−5^, OR = 1.12, 95% CI = 1.06–1.18). We further performed a combined meta-analysis for the *p*-values from these cohorts and our present data, as described by Glessner and Hakonarson [23]; we observe that rs3815087 reaches a genome wide significance of 1.90 × 10^−8^. The observed risk allele is associated with increased expression as per the GTex prediction tool for *PSORS1C1*.

Many studies have confirmed *PSORS1C1* on chromosome 6p21.3 to be a major locus for psoriasis susceptibility [24,25]. Case reports suggest a strong relationship between schizophrenia and psoriasis vulgaris, with the emergence of schizophrenia symptoms being accompanied by simultaneous exacerbation of psoriasis [26]. Another study using a nationwide population-based dataset in Taiwan demonstrated that patients with schizophrenia had an approximately 1.2-fold higher risk of concurrent psoriasis than the comparison cohort [27]. This was further supported by a recent meta-analysis comprising 6 million participants confirming that psoriasis is a major comorbid condition for schizophrenia and the risk of concurrent psoriasis significantly increases schizophrenia by 1.41 times [28]. One possible explanation for the positive association between schizophrenia and psoriasis is that inflammatory processes might be an important common pathogenic pathway leading to both schizophrenia and psoriasis.

In contrast, patients with schizophrenia have a strong negative correlation with another autoimmune condition, rheumatoid arthritis [29,30,31]. It has been hypothesized that they share a common immune etiology and that once an individual is affected by one of the diseases, then they become relatively immune to the other. It has been shown in GWAS studies and in our present study that the SNP rs3815087, which increases risk for schizophrenia, has a protective effect against rheumatoid arthritis (OR: 0.72, *p* = 1 × 10^−5^) [32]. This adds strength to our observation of strong positive association with schizophrenia.

A significant association with schizophrenia was also observed for rs9267487 at the allelic (*p* = 0.02) and genotypic (*p* = 0.003) levels. LD analysis revealed that this SNP was in strong linkage with the *TNFA* SNP rs361525 (r^2^ = 0.96 in controls). *TNFA* variant rs361525 has been reported to be significantly associated with schizophrenia in the same study population [11]. The SNP rs3815087 identified in this study may be the causative functional SNP, or it could be in LD with any other functional variants in the MHC region. A recent study reported polygenic susceptibilities to immune abnormalities and genetic overlap with severe mental phenotypes such as schizophrenia and psoriasis [33]. The implication that immune processes may interact with genetic risk to influence schizophrenia risk is consistent with several lines of evidence, implicating immune genes in schizophrenia susceptibility. Moreover, a selective risk for one autoimmune condition, psoriasis, and preventive advantage for the other autoimmune condition, rheumatoid arthritis, may indicate a complex interplay of immune and autoimmune function probably driven by the impact of Effector T (Teff) cells on the regulatory T (Tregs) cells. Tregs are reported to be hypofunctional in schizophrenia [34]. The Tregs are considered to be the main line of defense for autoimmune and inflammatory conditions and when both are compromised it is possible that autoimmunity may drive a subset of patients into schizophrenia. In Schizophrenia both are compromised. 

These observations are even more interesting from the point of view of treatment response as few studies have reported results of trials of immunosuppressive agents in schizophrenia. It has been shown that short-term treatment with azathioprine improved the psychiatric symptomatology in a subgroup of patients with schizophrenia [35]. Few other studies have set out to test well-defined immunosuppressive agents in schizophrenia. Even the antipsychotic drugs such as haloperidol and clozapine are highly immunosuppressive [36,37]. Emerging reports indicate that antipsychotics may also be involved in immune-mediated conditions, such as psoriatic rash [38]. Thus, investigating the immune related genes can hold significance not only from disease association point of view, but may also reflect on the role of antipsychotics or immunosuppressants in the treatment response and adverse effects. It is also important to note that medication in schizophrenia did elevate the Tregs which correlated with improvement in negative symptoms [39]. Therefore, precise understanding of the functional implication of MHC molecules and cytokines on the neurodevelopment with the impact of Teff cells on Tregs, may shed light on the role of comorbid conditions that may contribute to the pathogenesis of schizophrenia. 

## 5. Conclusions

Our finding support the role of MHC in schizophrenia and suggests a complex interplay with regulators of immune system such as cytokines and MHCs, which can act as potential but selective drivers for autoimmune hypothesis in schizophrenia. It also calls for identifying ethnic specific variants within the MHC loci which may help in providing deeper insight into comorbid conditions in schizophrenia. 

## Figures and Tables

**Table 1:** SNPs in the HLA region, functional effect, and the genotyping method.

SNo.	dbSNP rs ID	Locus	Position	Alleles	SNP Functional Effect	Nearby Gene	Distance (bp)	Genotyping Method
1	rs3130375	6p21.33	30429711	A/C	--	RPP21||LOC100129192	−7100||−43,009	KASPar assay
2	rs9368649	6p21.33	31046862	A/G	--	DPCR1||MUC21	−16,885||−12,612	KASPar assay
3	rs4713419	6p21.33	31101215	A/G	TFBS	MUC21||LOC729792	−35,561||−729	KASPar assay
4	rs3815087	6p21.33	31201566	G/A	Splicing regulation	PSORS1C1	10,964	KASPar assay
5	rs1819788	6p21.33	31367116	T/C	TFBS	HLA-C||HLA-B	−19,282||−62,512	KASPar assay
6	rs9266336	6p21.33	31439114	A/G	--	HLA-B||LOC729816	−6200||−2507	KASPar assay
7	rs9266718	6p21.33	31457767	C/T	--	LOC729816	−14,955	KASPar assay
8	rs9267415	6p21.33	31579809	A/G	--	MICB	5864	TaqMan allelic discrimination
9	rs9267487	6p21.33	31619329	C/T	TFBS	SNORD84||ATP6V1G2	−2395||−889	KASPar assay
10	rs1800629	6p21.33	31651010	A/G	TFBS	LTA||TNF	−933||−319	KASPar assay
11	rs361525	6p21.33	31651080	A/G	TFBS	LTA||TNF	−1003||−249	KASPar assay
12	rs3131296	6p21.32	32280971	T/C	TFBS	NOTCH4	10,373||18,851	KASPar assay
13	rs3130297	6p21.32	32306959	C/T	--	NOTCH4||C6orf10	−7137||−61,494	KASPar assay
14	rs9271850	6p21.32	32703038	A/G	TFBS	HLA-DRB1||HLA-DQA1	−37,479||−10,123	KASPar assay
15	rs9272219	6p21.32	32710247	G/T	TFBS	HLA-DRB1||HLA-DQA1	−44,688||−2914	KASPar assay

**Table 2:** Genotype and allele frequencies of the SNPs in MHC region with schizophrenia.

SNP		CC	AC	AA	P	C	A	P	OR (95% CI)
rs3130375	Control	242 (0.99)	2 (0.01)	0	0.12	486 (0.99)	2 (0.01)	0.03	5.0 (1.09–22.93)
	Case	239 (0.96)	8 (0.03)	1 (0.01)		486 (0.98)	10 (0.02)		
rs4713419		AA	AG	GG		A	G		
	Control	156 (0.64)	78 (0.32)	10 (0.04)	0.19	390 (0.8)	98 (0.2)	0.14	1.25 (0.93–1.70)
	Case	141 (0.57)	95 (0.38)	12 (0.05)		377 (0.76)	119 (0.24)		
rs3815087		CC	CT	TT		C	T		
	Control	149 (0.61)	90 (0.37)	5 (0.02)	1.58 × 10^−6^	388 (0.80)	100 (0.20)	1.14 × 10^−6^	2.0 (1.50–2.67)
	Case	112 (0.45)	103 (0.42)	33 * (0.13)		327 (0.66)	169 (0.34)		
rs1819788		AA	AG	GG		A	G		
	Control	234 (0.96)	10 (0.04)	0	0.41	478 (0.98)	10 (0.02)	0.33	1.49 (0.66–3.35)
	Case	234 (0.94)	13 (0.05)	1 (0.004)		481 (0.97)	15 (0.03)		
rs9266336		GG	AG	AA		G	A		
	Control	111 (0.45)	134 (0.55)	0	0.92	356 (0.73)	132 (0.27)	0.99	0.99 (0.75–1.32)
	Case	114 (0.46)	134 (0.54)	0		362 (0.73)	134 (0.27)		
rs9266718		TT	CT	CC		T	C		
	Control	156 (0.64)	76 (0.31)	12 (0.05)	0.23	390 (0.80)	98 (0.20)	0.73	1.05 (0.77–1.43)
	Case	151 (0.61)	90 (0.36)	7 (0.03)		392 (0.79)	104 (0.21)		
rs9267415		GG	AG	AA		G	A		
	Control	85 (0.35)	110 (0.45)	49 (0.20)	0.27	280 (0.57)	208 (0.43)	0.13	1.2 (0.94–1.57)
	Case	97 (0.39)	114 (0.46)	37 (0.15)		308 (0.62)	188 (0.38)		
rs9267487		TT	CT	CC		T	C		
	Control	217 (0.89)	25 (0.10)	2 (0.01)	0.003	459 (0.94)	29 (0.06)	0.03	1.66 (1.02–2.68)
	Case	201 (0.81)	47 (0.19)	0.00		449 (0.90)	47 (0.10)		
rs1800629		GG	AG	AA		G	A		
	Control	192 (0.78)	51 (0.21)	2 (0.01)	0.09	434 (0.89)	54 (0.11)	0.62	1.11 (0.74–1.66)
	Case	203 (0.82)	40 (0.16)	5 (0.02)		446 (0.90)	50 (0.10)		
rs361525		GG	AG	AA		G	A		
	Control	217 (0.89)	25 (0.10)	2 (0.01)	0.019	459 (0.94)	29 (0.06)	0.018	1.77 (1.10–2.85)
	Case	199 (0.80)	48 (0.19)	1 (0.01)		446 (0.90)	50 (0.10)		
rs3131296		GG	AG	AA		G	A		
	Control	207 (0.85)	35 (0.14)	2 (0.01)	0.51	449 (0.92)	39 (0.08)	0.47	1.17 (0.75–1.84)
	Case	207 (0.83)	36 (0.15)	5 (0.02)		450 (0.91)	46 (0.09)		
rs9271850		AA	AG	GG		A	G		
	Control	160 (0.66)	66 (0.27)	18 (0.07)	0.26	386 (0.79)	102 (0.21)	0.68	1.06 (0.78–1.44)
	Case	151 (0.61)	85 (0.34)	12 (0.05)		387 (0.78)	109 (0.22)		
rs9272219		GG	GT	TT		G	T		
	Control	191 (0.78)	46 (0.19)	7 (0.03)	0.65	428 (0.88)	60 (0.12)	0.70	1.07 (0.74–1.56)
	Case	187 (0.75)	57 (0.23)	4 (0.02)		431 (0.87)	65 (0.13)		

* Recessive model (TT vs. CT + CC) *p* = 1.17 × 10^−6^, OR = 7.3, 95% CI 2.81–19.13.

## Data Availability

Data will be available on request.
